# 

**Published:** 1922-02

**Authors:** 


### HOSPITAL and HEALTH

REVIEW

*Established 1886* No. 1847. *Vol. LXXI.*

*New Series.* Vol. 1. No. 5 *February* 1922 Price Sixpence

CONTENTS.

| | PAGE |
|---|---|
| Notes and Comments . . . . . . . . | 121 |
| The A B C of Vitamins . . . . . . . . — Harold Scurfield, M.D., D.P.H. | 125 |
| Hospitals and Unemployment . . . . . . | 126 |
| The Late Sir Perceval A. Nairne . . . . | 127 |
| King Edward's Hospital Fund . . . . . . | 128 |
| Notable Epidemics—IV., The Influenza of 1918-1919 . . . . . . . . . . . — A. T. Nankivell, M.D., D.P.H. | 129 |
| The Review of the Month . . . . . . | 131 |
| The Fundamental Biological Factors Underlying the Incidence of Tuberculosis . . — R. J. Ewart, M.D., D.Sc., F.R.C.S. | 133 |
| Notes from the Conference of Educational Associations . . . . . . . . . | 136 |
| News in Brief . . . . . . . . . . | 138 |
| Diphtheria in Bristol, 1921 . . . . . . | 139 |
| Public Health Legislation in 1921 . . . . | 141 |
| Passing Events and Topics . . . . . . | 143 |
| Reviews; Our Prize Leaflets; Correspondence | 146-9 |

